# Forage intake and digesta kinetics of lactating beef cattle differing in feed efficiency while grazing Idaho rangelands

**DOI:** 10.1093/jas/skaf429

**Published:** 2025-12-13

**Authors:** James E Sprinkle, Carmen M Willmore, Melinda J Ellison, John B Hall, Ronald M Lewis, Douglas R Tolleson, David M Jaramillo

**Affiliations:** Department of Animal, Veterinary and Food Sciences, University of Idaho, Moscow, ID 83844; University of Idaho Nancy M. Cummings Research, Extension & Education Center, Carmen, ID 83462; Bingham County Extension Office, University of Idaho, Blackfoot, ID 83221; Department of Animal, Veterinary and Food Sciences, University of Idaho, Moscow, ID 83844; University of Idaho Nancy M. Cummings Research, Extension & Education Center, Carmen, ID 83462; Department of Animal, Veterinary and Food Sciences, University of Idaho, Moscow, ID 83844; University of Idaho Nancy M. Cummings Research, Extension & Education Center, Carmen, ID 83462; Department of Animal Science, University of Nebraska-Lincoln, Lincoln, NE 68583; Texas A&M AgriLife Research-Sonora, Sonora, TX 76950; US Dairy Forage Research Center, USDA-Agricultural Research Service, Marshfield, WI 54449

**Keywords:** beef cattle, digesta kinetics, forage intake, grazing behavior, rangeland, residual feed intake

## Abstract

The objective was to determine if 2-yr old cows differing in residual feed intake (RFI) would differ in forage intake and digesta kinetics. Previously classified 2-yr-old lactating Angus × Hereford cows (12 efficient, [EFF]; 12 inefficient, [INE]) were given a pulse dose of an alkane marker and outfitted with grazing collars. Fecal samples were then collected repeatedly over 4 d in June and August. Daily digestible organic matter (DOM) and crude protein (CP) were estimated from fecal near infrared spectroscopy. Data were analyzed by fitting a non-linear digesta kinetics model to individual cows. A mixed model with RFI treatment, period, and their interaction as fixed effects, and cow within treatment as a repeated random effect, was fitted to the combined kinetics data. There were no treatment differences (*P *> 0.05) between EFF and INE cows for the variables measured, but period differences were present (*P *< 0.05). Fecal output tended to increase from spring to summer (*P *= 0.08) and for INE cattle from spring to summer (*P *= 0.06) but EFF cattle did not differ from spring to summer (*P *= 0.59). Total marker residence time in the gastrointestinal tract (RTG) was 38.3 and 40.5 ± 1.2 h for INE and EFF cows, respectively in June, and 44.0 and 42.9 ± 1.2 h for INE and EFF cows in August, increasing (*P *< 0.001) for INE and tending to increase for EFF (*P *= 0.097). Period RTG was 39.4 vs. 43.4 ± 0.8 h for June vs. August (*P *< 0.01). Dry matter intake (DMI) in June was 15.6 ± 1.15 kg for INE cows and 17.6 ± 1.10 kg for EFF cows (*P *= 0.22). August DMI was 17.0 and 16.3 ± 1.10 kg for INE and EFF cows, respectively (*P *= 0.67). The DOM was 58.9% vs. 53.8% ± 0.36% for June vs. August (*P *< 0.0001) and CP was 15.0% ± 0.23% vs. 14.2% ± 0.22% (*P *< 0.05). The CP in the diet declined from spring to summer for EFF cattle (*P *< 0.05), but not for INE cattle (*P *= 0.22). The compartmental mass of undigested dry matter (fill) was 6.6 vs. 8.6 ± 0.42 kg for June vs. August (*P *< 0.01). Bite rates increased from spring to summer (*P *< 0.05) and for EFF cattle (*P *< 0.05), but not INE cattle (*P *= 0.52). Grazing time tended to increase from spring to summer (*P *= 0.06), and for INE cattle from spring to summer (*P *= 0.07), but not for EFF cattle (*P *= 0.41). Reasonable, though slightly inflated, estimates of DMI with the pulse dose procedure were obtained, but differences in DMI for RFI in a feedlot setting did not translate to a range setting.

## Teaser

The ability to select an animal from birth that best suits an environment based on efficiency would be a significant improvement to the cattle industry. But have we found a way to measure efficiency of intake in a range setting?

## Introduction

The ability to estimate individual animal intake on rangeland has become increasingly important to the cattle industry in the West as it transitions to more management intensive grazing (Galyean and Gunter, 2016).

Based upon previous research and the interaction of genotype and environment, it is important to fully evaluate how low and high residual feed intake (RFI) cows perform in different environments to determine their “fitness” for those environments. A cow that excels in a favorable environment may not perform similarly in a restrictive environment. Previously, other researchers have made efforts to categorize beef cattle that are efficient on western rangelands (Kress and MacNeil, 1999). In this study they found that cows that excelled in Havre, MT, US at higher elevations did not perform comparably when they were moved to Miles City, MT, US with reduced rainfall and elevation. In another assessment of the effect diet may have on a cow’s production, an extensive study looked at nine different breeds of cattle over a 5-yr period (Jenkins and Ferrell, 1994). This study analyzed four different levels of feed and its impact on kg of calf weaned, kg of feed consumed, and number of cows exposed for breeding. They found that cattle that consumed more when feed resources were abundant did produce more (g calf weaned · kg DM^−1^ ·cow exposed^−1^). However, when feed resources were restricted, the larger cattle were less efficient, and the more moderate sized cattle performed favorably.

Feed efficiency research particularly has become more important with growing concerns about production efficiency within the cow herd. However, most feed efficiency testing has only been performed in a dry lot setting which does not have the same relationship and effect on the animals being tested as in a grazing environment. Few studies have seen forage intake for divergently ranked RFI cattle that was determined in a feedlot setting carryover to a pasture/range setting. A study conducted in Alberta, Canada on improved fertilized pasture found that yearling heifers with previously determined low RFI in a feedlot setting ate less dry matter (DM) than did the high RFI heifers (Manafiazar et al., 2015). Research in Australia performed with cows on improved pasture (Herd et al. 1998) determined that feedlot efficient cows had improved nutrient partitioning but with no reduction in forage intake when compared to feedlot inefficient cows. A study in Brazil with Nellore male and female yearling cattle did not find any difference in pasture forage intake among previously feedlot classified low, medium, and high RFI cattle (Oliveira et al., 2018). Two studies conducted in Ireland (Lawrence et al., 2012, 2013) found that RFI was positively associated with silage dry matter intake in the feedlot but not grazed forage for the same experimental animals. Other mechanisms, such as rumen fill of lower quality forage, may need to be examined more fully. The sparse studies examining forage intake of divergently ranked RFI grazing cattle have not evaluated how these animals compared with respect to the mechanisms of forage digestion (digesta kinetics).

The use of fecal near infrared spectroscopy (NIRS) analyses to estimate forage digestibility for grazing beef cattle can provide individual estimates (each cow is a sample unit) rather than a single group estimate (subset of cannulated or fistulated cattle; hand-plucked forage) of digestibility. With this approach, adaptive grazing mechanisms for cattle differing in biological efficiency can be investigated. The forage selectivity of divergently ranked beef cattle (such as by RFI) can be evaluated at time periods when forage quality is at its peak (e.g. springtime) or as it is declining (e.g. late summer). However, the use of NIRS fecal sampling to determine forage digestibility has not always been embraced, particularly in the Northern Great Plains (Harty and Olson, 2018; Harty and Jenkins, 2019). Specifically, these authors concluded that fecal NIRS predictions of nutritional quality for consumed forage for beef cattle in the Northern Great Plains were not useful since they failed to generate a perfect 1:1 prediction curve. The authors also offered criticism of the NUTBAL prediction software that is often used with NIRS fecal sampling.

The objective of this study was to determine if 2-yr old cows differing in RFI, differed in forage intake, digesta kinetics (forage passage rate, gastrointestinal tract size, gastrointestinal tract residence time), energy intake, and harvest efficiency during spring or summer while grazing native rangeland. A secondary objective was to compare spring and summer forage quality as estimated by fecal NIRS samples to forage samples analyzed by traditional laboratory wet chemistry. We hypothesized that cows differing in RFI would exhibit differences in forage intake and subsequent digestive kinetic processes while grazing a common range environment.

## Materials and Methods

All procedures were approved by the University of Idaho Animal Care and Use Committee (IACUC # 2015-44). Animal husbandry, management, and handling procedures in the research environment were in accordance with the Ag Guide (2010).

### Range sites

This trial was conducted over spring and summer grazing periods in 2016 at the Rinker Rock Creek Ranch (RRCR) located about 18 km southwest of Hailey Idaho (114° 23.509’ W 43° 23.426’ N). The ranch is described more fully at https://www.uidaho.edu/cnr/rangeland-center/rock-creek but consists of 4,209 ha private land and 4,452 ha of public land, the majority of the public land being administered by the Bureau of Land Management. Upland sagebrush-steppe pastures were grazed by approximately 160 cows from 14 June to 4 July in 2016 in a 909-ha pasture (1,463 to 1,646 m elevation; slopes up to 68% but predominantly 0% to 15%) and from 2 August to 25 August in a 1,345-ha pasture (1,510 to 1,726 m elevation; slopes up to 45% but predominantly 5% to 25%).

Dominant ecological sites (provisional) for pastures grazed earlier in the grazing season were located within the Elkcreek-Polecreek (25%), Laurentzen-Muleshoe (40%), and Winu-Gaib (13%) soil complexes and included R010AY004ID, R010AY001ID, R010AY008ID, and R010AY021ID. Dominant ecological sites (provisional) for pastures grazed in late summer were within the Moonstone-Earcree soils association (89%) and included R010AY009ID and R010AY008ID. These descriptions are available from the NRCS Web Soil Survey https://websoilsurvey.sc.egov.usda.gov/App/HomePage.htm.

The mean annual precipitation (1981 to 2010) near the research sites at the airport in Hailey, Idaho (114° 18.171'W 43° 30.448'N, elevation 1,617 m) is 341 mm with 48% falling during April through September. Pastures are dominated by mountain big sagebrush (*Artemisa tridentate Nutt. ssp. vaseyana* [Rydb.] Beetle) with subdominant shrub species including antelope bitterbrush (*Purshia tridentata* [Pursh] DC.) and rabbitbrush (*Chrysothamnus* Nutt.). Prominent half-shrubs include sulphur-flower buckwheat (*Eriogonum umbellatum* Torr.). Dominant perennial grasses include Great Basin wildrye (*Leymus cinereus* [Scribn. & Merr.] A. Löve), Columbia needlegrass (*Achnatherum nelsonii* [Scribn.] Barkworth ssp. Nelsonii), Idaho fescue (*Festuca idahoensis* Elmer), sandberg bluegrass (*Poa secunda* Presl), prairie junegrass (*Koeleria macrantha* [Ledeb.] Schult.), bluebunch wheatgrass (*Pseudoroegneria Spicata* [Pursh] A. Löve ssp. spicata), and bottlebrush squirreltail (*Elymus elymoides* [Raf.] Swezey ssp. *Elymoides*). The dominant annual grass is cheatgrass (*Bromus tectorum*). The dominant forbs are arrowleaf balsamroot (*Balsamorhiza sagittata* [Pursh] Nutt.) and lupine (*Lupinus* L. spp).

### Forage production and nutritive value

In 2016, forage production was estimated at the beginning of each grazing period by hand clipping 20 randomized 0.16 m^2^ quadrats in an area representative of the experimental pastures. All perennial and annual graminoids rooted within the quadrat frame within the sampled areas were clipped to ground level and dried for 48 to 71 h at 65 °C. Palatable half shrubs and edible forbs were clipped separately and analyzed as browse. The majority of browse consisted of sulfur-flower buckwheat and only the current year’s plant leaders were clipped for this plant. Sagebrush canopy was not sampled for production.

Crude protein ([CP]; Padmore, 1990a, 1990b; Gavlak, et al., 1996; Miller et al., 1997) was determined on replicate samples (*n *= 5 clipped plots/replicate) of clipped forage by a commercial lab (Ward Laboratories, Inc., Kearney, NE). Forage digestibility of the clipped forage samples at the same lab was estimated *in vitro* from acid detergent fiber using the Ankom 200/220 Fiber Analyzer (Ankom Co., Macedon, NY) and following the procedures of Mertens (1992).

### RFI determination and cow allocation

Yearling heifers (*n *= 160) at the Nancy M Cummings Research Extension and Education Center (NMCREEC), Carmen, Idaho were evaluated for RFI over a 70-d test period on an 80% roughage diet as outlined by Hall et al. (2015) with an added covariate being introduced into the regression model that adjusted the individual RFI scores for 12^th^ rib backfat at the conclusion of the feeding period (Basarab et al., 2011). Individual feed intakes were obtained using GrowSafe (GrowSafe Systems Ltd, Airdire, Alberta, Canada; now Vytelle SENSE, Lenexa, KS, USA) equipment at NMCREEC and heifers were classified as either average, efficient (EFF, negative RFI), or inefficient (INE, high RFI). The heifer RFI scores were categorized by their standard deviation according to the contemporary mean. Heifers classified as EFF had RFI ≤ 0.5 standard deviations below the mean and those classified as INE had RFI ≥ 0.5 standard deviations above the mean. These heifers were developed at NMCREEC the first year for artificial insemination breeding and grazed irrigated pasture.

The following year, a cohort of 2-yr-old lactating cows (*n *= 24; 12 EFF, 12 INE) were selected to participate in this forage intake trial on native range. The average RFI standard deviations of EFF and INE cows were −0.91 ± 0.068 and 0.84 ± 0.068, respectively. Following artificial insemination breeding (day 94.9 ± 2.2 of lactation) at NMCREEC, cows and calves were transported by truck on 19 May 2016, for 4 h to RRCR for summer grazing on native range. Cows were acclimated to the range setting 30 d prior to the start of the first pulse-dose forage intake trial. Cattle were evaluated in both mid-lactation (133 ± 2.7 d) and late-lactation (182 ± 2.7 d) as forage quality was near its traditional peak and as it was declining, respectively.

### Cow measurements

Cow weights and body condition score (BCS; 1 to 9, 9 = fattest; Richards et al., 1986) were obtained 6 to 7 d (single measurement) prior to shipping to RRCR and again on 1 August 2016. Any shrink was incidental to the process of gathering and holding cattle prior to weighing and BCS scoring. The amount of hold time away from grazing prior to weighing was greater in August (8.5 to 10.5 h) than in the spring (4 to 6 h) due to the necessity of obtaining additional research data for another study (Sprinkle et al., 2021a).

#### Pulse-dose marker preparation and forage intake sampling

Preceding allocation to the summer grazing pastures, 24 two-yr-old Hereford×Angus were outfitted with grazing logger halters and separated from the rest of the herd for 5 d by cordoning off a section (16.2 ha for spring; 33.6 ha for summer) of the upland rangeland pastures using temporary electric fence. With this pulse-dose forage intake study, frequent fecal samples were required, and the smaller pastures made it easier to retrieve repeated fecal samples from the free ranging cattle on these upland pastures. Additionally, observational data on these same cattle were collected by multiple observers to calibrate halter mounted electronic equipment used to obtain grazing behavior data (Sprinkle et al., 2021a, 2021b).

Since we desired to obtain forage intake for free ranging beef cattle on native rangeland pastures, we were unwilling to gather cattle daily to administer an alkane dose. Doing so would unduly disturb grazing activity and bias forage intake among our treatment groups. For example, Sprinkle et al. (2021c) reported that the bite rate for cattle on dormant sagebrush steppe pastures increased (*P *< 0.001) from 49 bites/min the day before corralling to 61 bites/min the day of corralling following release into the pastures. On the day following corralling, the bite rate returned to normal at 50 bites/min. Therefore, to minimize disturbance, our only option was to use a pulse dose procedure where the cattle only needed to be gathered the afternoon preceding the start of the 4-d sample collection period. At the time of our study, continuing to today, there are no commercially available constant release devices that dispense an external marker over a period of days. There was a commercially available alkane constant release device available in the early 2000’s, but that product was discontinued in 2008 (Dove, 2010).

Mayes and Dove (2000) suggested that alkane markers are more associated with the fiber portion of the digesta, which is important for us to determine the flow of consumed forage through the digestive tract. Giraldez et al. (2004) tested several different chain lengths of alkanes coupled with five different carrier matrices, and they concluded that the C_32_ alkane that was absorbed onto shredded filter paper was superior to the other chain lengths and carrier matrices used.

On the afternoon preceding (with average time = 1458 h for June 2016) the first day of fecal sampling by approximately 15 h, a long chain (C_32_) alkane (dotriacontane, Acros Organics, Fair Lawn, NJ) was administered as an external marker in a pulse- dose fashion (Sprinkle et al., 2000; Giraldez et al., 2004). Alkane boluses were prepared in 8 individual batches using 35.39 g of C_32_ alkane in 509 mL n-heptane (Fisher Scientific, Fair Lawn, NJ) for each batch. The alkane heptane mixture was heated to 80 °C for 15 min or until alkanes were fully dissolved. The mixture was then absorbed onto 20 preheated (100 °C for 30 min) sheets of 450 × 330 mm (258.39 g) Whatman No. 1 filter paper (Sigma-Aldrich, Saint Louis, MO) contained within an aluminum tray following the procedures of Mayes et al. (1986) and Keli et al. (2008). The filter paper sheets were then hang-dried for 4 h. Since the negative pressure of the fume hood was insufficient to completely dry the alkane impregnated filter sheets, a blow hair dryer was used to complete the process. After visual and tactile assessments that the filter paper was dry, the cooled paper was then shredded into 2.3 × 0.8 cm strips using an 8-sheet Crosscut Credit Card Shredder (Bonsen, Flowery Branch, GA). The shredded strips were then loaded into gelatin boluses (Size Su07, Torpac, Inc., Fairfield, NJ) at a rate of 6.5 g of paper per bolus with each bolus containing 756 mg alkane. Each animal was given six boluses via a multi-dose balling gun (Model C07856, Nasco, Fort Atkinson, WI). The total weight of boluses was 39 g creating an alkane dose of 4.54 g.

The dose concentration of the alkane boluses was validated by later laboratory analyses of multiple (*n *= 20) intact boluses by the US Dairy Forage Research Center in Marshfield, WI. The quantitative recovery of the n-alkane within these boluses was conducted by soaking the shredded strips with heptane under heating and decanting the alkane-heptane solution into a pre-weighed beaker, as described by Jaramillo et al. (2025). A sample air concentrator (Cole-Parmer SC-200, Cole-Parmer North America, Vernon Hills, IL) was used to elude the heptane and the recovered alkane was weighed. The average C_32_ content of each bolus was 756 ± 40 mg.

At the time of pulse-dosing cattle, and immediately prior to administering the 4.54 g pulse dose of alkanes, fecal samples were obtained rectally from each cow to analyze for background concentrations of C_32_ alkane in the forage.

**Figure 1.**
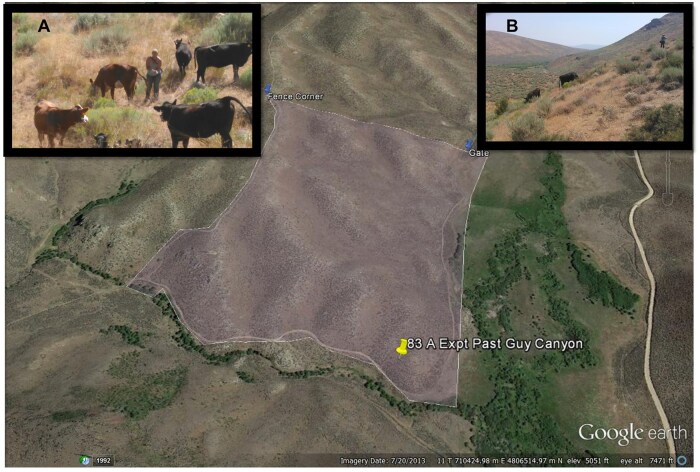
Experimental 34-ha pasture used in August 2016 for free-ranging beef cattle with forage intake determined by recovery of fecal samples from an alkane pulse dose procedure. Inset left (A), cattle were used to humans and could be closely approached. Inset right (B), the experimental sagebrush steppe pasture contained significant terrain.

The first sampling occurred 14 to 17 June 2016, during mid-lactation and a second sample period occurred 2 to 5 August 2016, during late-lactation. Fecal samples were collected over a 4-d period as cows were defecating. The cows were acclimated to people and could be closely approached within 6-m without causing disturbance (Figure 1). In addition to these two sampling periods, a forage intake validation trial was conducted from 10 to15 December 2016, at NMCREEC using the GrowSafe facility and will be described later.

Allowing for at least a 12 h lag period for the alkane marker to achieve mixing, frequent sampling of fecal samples by multiple observers (4 to 6) occurred over the next 4 d (Sprinkle et al., 2000) while all cows were free ranging in the temporary 16.2 and 33.6 ha pastures enclosed by an electric fence. Because the fecal excretion pattern of the alkanes was a logarithmic curve, fecal samples were obtained whenever cows were seen defecating the first day (to fit the logarithmic curve) starting at daybreak. Sampling continued 3X/d for the second and third day (early morning, mid-day, mid to late afternoon), and 2X/d on the final day of fecal sampling (early morning, mid-day to early afternoon). As cows dropped their dung in the experimental pastures, observers obtained samples with a plastic baggie, labeled it with cow ID, date, and time, and put it in a cooler for later freezing.

Fecal samples were frozen in a conventional freezer at −18 °C. Samples were then dried at 60 °C and ground through a 1-mm screen. Ash concentration of fecal samples was determined by a commercial lab (Ward Laboratories, Inc., Kearney, NE). Alkane laboratory analyses followed the protocol described in Dove and Mayes (2006) and were conducted at the University of Nebraska-Lincoln (Lincoln, NE) on all repeated fecal samples. The background fecal samples were analyzed by the US Dairy Forage Research Center (Marshfield, WI) following the same procedure described above. Extractions were performed on each fecal sample in duplicate and the intra-assay CV associated with these repeat analyses at the University of Nebraska-Lincoln averaged 4.11% over 1,406 samples.

Quantification of alkane concentrations in the fecal samples and alkane boluses used were conducted by gas chromatography. The gas chromatography at the University of Nebraska-Lincoln followed procedures as described by Lewis et al. (2016). Gas chromatography analyses at the US Dairy Research center was performed using a DeltaQ isotope ratio mass spectrometer (Thermo Fisher, Bremen, Germany) coupled to a Trace1310 gas chromatograph (Thermo Fischer, Bremen, Germany). The gas chromatrograph was equipped with a fused silica capillary column (30 m × 0.25 mm i.d., 0.10 μm film thickness). Helium was used as the carrier gas, with injector and detector temperatures set at 210 °C. Oven temperature was initially set at 210 °C, ramped up at 15 °C/min to 320 °C, and held for 5 min. Peak areas were quantified using QTegra software, and alkane concentrations were calculated based on the internal standard, sample organic matter weight, and response factors (SRFi).

With the results of the alkane concentrations in fecal samples, fecal output was determined by dilution of the dose and ultimately forage intake as calculated by dividing the fecal output by (1-digestibility) by an estimation of digestibility for each cow with fecal NIRS analyses (Lyons and Stuth, 1991, 1992; Lyons et al., 1993). The digestibility and crude protein estimates for each cow were determined daily over 3 d with the first fecal sample of each day being analyzed by fecal NIRS at the Texas A & M AgriLife Sonora Research Station (Sonora, TX).

#### Validation trial

A feedlot validation trial was conducted using four nonlactating mature cows in a GrowSafe unit at NMCREEC in December 2016. Total fecal collection was preceded by a 10-d adaptation period in which all cows were offered *ad libitum* access to chopped orchardgrass-alfalfa hay in a concrete bunk. Following this adaptation period, each cow was placed on concrete in individual pens (103 sq. m) with her own GrowSafe feed bunk and had another cow in an adjacent pen that she could see and smell. Cows were administered a 4.54 g pulse dose of alkanes at approximately 1715 h on 10 December, and all fecal material was obtained from 0000 h on 11 December to 0800 h on 15 December. Fecal patties that dropped on the concrete from each cow were obtained at multiple intervals during each 24 h period with a scoop shovel. Each time a cow was observed defecating, a fecal grab sample was obtained for later alkane analysis. Wet samples of the daily fecal output were weighed twice daily, and dry matter was determined on a sub-sample of each multiple sample. Total feed intake of a 57% Total Digestible Nutrients (TDN) chopped forage diet (validated by Ward Labs, Kearney, NE) was determined with the GrowSafe units. The fecal output estimated from the alkane pulse dose procedure was converted (Merchen, 1988) to day matter intake (DMI) by dividing the fecal output by the indigestibility of the forage (1—TDN). Multiple grab samples of fecal patties were obtained over 4 d to obtain the concentration of alkanes in the fecal samples. Predicted forage intake from the time the alkane pulse dose was active from approximately 0200 to 0400 h on 11 December until 2300 h on 13 December was then compared to the actual GrowSafe average DMI from 11 to 13 December.

#### Grazing time

All cows used in the rangeland study also carried a customized grazing halter containing both a global positioning system (GPS) logger and a 3-axis accelerometer as described by Sprinkle et al. (2021a). With the accelerometer sensor, we were able to obtain daily estimates of grazing time over 5 s intervals for each cow in this study.

#### Bite rate

Focal sampling for bite rate (BR, bites/min) was conducted on single animals (Sprinkle et al., 2000) over the last 3 d of each sample period. Observations were recorded during either the AM or PM grazing bouts for approximately 10 to 15 min by 2 observers. Cows watched rotated among observers on alternate days or with duplicate observations on the same day to help alleviate variation among observers. At least 4 replicate samples per observation period were acquired whenever possible. Beginning and ending times for each replicate were recorded in the field on a tablet computer using a spreadsheet with an integrated timestamp. Sometimes (3%) cattle commenced resting, walking to water, or ruminating during an observed grazing bout, so it was not always possible to obtain multiple sample replicates of 4 or greater during the grazing observation period. Bite rate frequency data were averaged over each observation period. Any BR average with less than 3 reps was deleted.

#### Calculations

As fecal samples were obtained from free ranging cattle in this study, we discovered that some of the observers scooped up some soil resulting in elevated ash (21.2% average). As a comparison, the average ash concentrations for the fecal samples recovered during the feedlot validation trial was 15.6%. Therefore, forage intake will be reported as DMI on a dry matter digestibility basis instead of an OM basis. For those digesta kinetics values reported which rely on some estimate of OM of the fecal samples (compartmental mass of undigested OM in the digestive system [CMS]; energy intake [EI]; consumed forage metabolizable energy [ME]; and harvest efficiency [HE]), default fecal ash values were set to not exceed 15% (Meissner and Paulsmeier, 1995) for back calculations to fecal organic matter output. Since digestibility obtained from fecal NIRS was reported as digestible organic matter (DOM), 5% was added to these values to obtain TDN (Huston and Pinchak, 1991). With the combination of grazing time and forage intake, harvest efficiency was determined by:


$$\frac{\mathrm{grams}OM\mathrm{intake}}{kg\mathrm{cow}\mathrm{weight}}÷\mathrm{average}\mathrm{total}\mathrm{grazing}\min=\frac{\mathrm{grams}OM\mathrm{intake}}{kg\;BW\;x\mathrm{minute}of\mathrm{grazing}}$$

Harvest rate was calculated by:


$$\frac{g\;\mathrm{DMI}/d}{\min/d\;\mathrm{grazing}\;\mathrm{time}\;x\;\mathrm{bites}/\min\;\mathrm{grazing}\;\mathrm{time}}=\frac{g\;\mathrm{DMI}}{\mathrm{bite}}$$

Energy intake was expressed as:


$$\frac{kg\;OM\;\mathrm{intake}/d\;x\;\mathrm{Mcal}ME/kg\;OM\;\mathrm{intake}\;x1000\;\mathrm{kcal}/\mathrm{Mcal}}{kg\;BW^{0.75}}=\frac{\mathrm{kcal}\;ME/d}{kg\;BW^{0.75}}$$

where kcal was estimated (Huston and Pinchak, 1991) from fecal NIRS predicted DOM kilocalories = (((average DOM × 4.6) × 0.82) × 1000) and metabolic body weight was obtained by kg cow weight raised to the 0.75 power.

### Statistical analysis

Modeling digesta kinetics involves fitting a nonlinear logarithmic recovery curve of an administered external marker in the feces of each animal. The primary rate parameters related to digesta kinetics (λ_1_, k_2_, τ, and C_2_) were estimated for each cow using the two-compartment, age-dependent, age-independent model (G2 → G1 → τ → 0) of digesta flow (Ellis et al., 1994). The primary nonlinear rate parameters related to digesta kinetics including k_2_ (passage rate from rumen), λ_1_ (lambda; escape turnover time from the first mixing compartment for forage particles), τ (tau; time delay of dosed alkane to first appearance in feces), and C_2_ (concentration of marker in digesta in slower turnover compartment assuming instantaneous mixing) are explained more fully in Sprinkle et al. (2000). Each experimental animal was run through this NLIN procedure in SAS (vs. 9.4, SAS Inst., Inc., Cary, NC) to obtain solutions for each unique curve of recovery as described by Ellis et al. (1994). As nonlinear solutions were obtained, the maximum allowable passage rate (k_2_) was capped at ≤ 0.061 for this rangeland pasture study. We did not apply this constraint to the validation study, which had passage rates varying from 4.91%/h to 6.51%/h for the four cows in that trial. Although reported passage rates for beef cattle on forage diets have varied from 1.5%/h to 7.8%/h (rare earth and Cr_2_O_3_ external markers; Seo et al., 2006), passage rates more commonly reported for grazing animals are often in the 3%/h to 4%/h range (Sprinkle et al., 2000; Schlecht et al., 2007). In one trial that evaluated passage rate for shredded paper C_32_ boluses such as used in this study, the particulate passage rate was 8.1%/h for goats (Giraldez et al., 2004), which usually have greater passage rates than do cattle (Schlecht et al., 2007). Therefore, we felt justified in establishing an *a priori* maximal passage rate of 6.1%/h for this study as nonlinear estimates of digesta kinetics were generated. To help nonlinear analyses to converge, preset parameter estimates for each cow were fine-tuned with multiple runs to achieve the lowest possible mean square error for the model. As the models were fine-tuned, the number of iterations for the model prior to convergence decreased. Bound statements we established required that λ_1_, k_2_, τ, and C_2_ solutions all be greater than zero, and k_2_ ≤ 0.061. When some logarithmic curves were more difficult to converge, it sometimes required adjacent fecal samples (2 h or less time difference) to be deleted. When alkane fecal concentrations had very similar values and were close in proximity, the logarithmic curve was forced sideways, inhibiting convergence of the model. Whenever we encountered some alkane concentrations that clearly did not fit the logarithmic curve (such as big dips in alkane concentrations out of sequence with the curve), those points were also deleted. All total, 22 individual alkane concentrations over all 24 cows in this study were deleted from the two sample periods (3.74% of 589 total fecal rangeland samples). Additionally, when curves proved difficult to converge, we increased the offset convergence value from our standard 0.1 to 0.4 or 06. The NLIN optimization method used was the Marquardt method and the step size search method used was the golden method. Coding and example datasets for this NLIN procedure in SAS can be obtained from the address shown in the section immediately below. Solutions for the parameters generated by these NLIN procedures were next used for calculations of compartmental mass, residence times, turnover times, and daily output of undigested dry matter.

Data results and the resulting calculations for digesta flow from the NLIN procedure included C_2_, lambda, tau, passage rate, fecal output, DMI, EI, percentage of body weight intake, HE, CM1 (compartmental mass of undigested OM in the age-dependent first compartment of digestive system), and CM2 (compartmental mass of undigested OM in the age-independent second compartment of digestive system). Additional, derived values were CMS (CM1 + CM2), MCRT1 (residence time of marker in the slower turnover mixing compartment CM2), MCRT2 (residence time of marker in the faster turnover mixing compartment CM1), MCRTS (MCRT1 + MCRT2), RTG (gastrointestinal tract residence time or equivalent to MCRTS + tau), and CF2 (flux of undigested OM from CM2). These response variables were analyzed by mixed model procedures (SAS vs. 9.4, SAS Inst., Inc., Cary, NC) with RFI treatment, period (spring or summer), and the interaction as fixed effects, with cow within treatment as a repeated random effect. The NIRS crude protein, kcal, and DOM values, grazing time, cow weight, and BCS data were analyzed using the same statistical model.

Replicate forage samples were analyzed for forage quality by mixed model procedures with pasture, type (grass or browse), and pasture × type × season as fixed effects and with replicate within pasture × type as a random effect.

Statistical differences in least square means were evaluated using the pdmix800.sas macro as originally described by Saxton (1998).

### Statistical programming coding for nonlinear analysis of pulse dose marker presence

As we attempted to run the published nonlinear code (Moore et al., 1992) for fitting the digesta kinetics two-compartment, age-dependent, age-independent model (Ellis et al., 1994), we were unsuccessful in getting the code to execute. Other scientists have reported similar problems (personal communication, Dr Stacey Gunter, USDA Research Center, Woodward, OK). We consulted one other source for coding, the Texas Tech University lab manual authored by Galyean (2010), and then commenced troubleshooting code. After spending a week of troubleshooting, we were successful in executing code. We have included example datasets and the corrected SAS data coding on the following Open Source Framework cloud data sharing platform https://osf.io/rfzv5/?view_only=89684cf6453245e4990859adf6745325.

## Results and Discussion

### Alkane pulse dose validation trial

**Figure 2.**
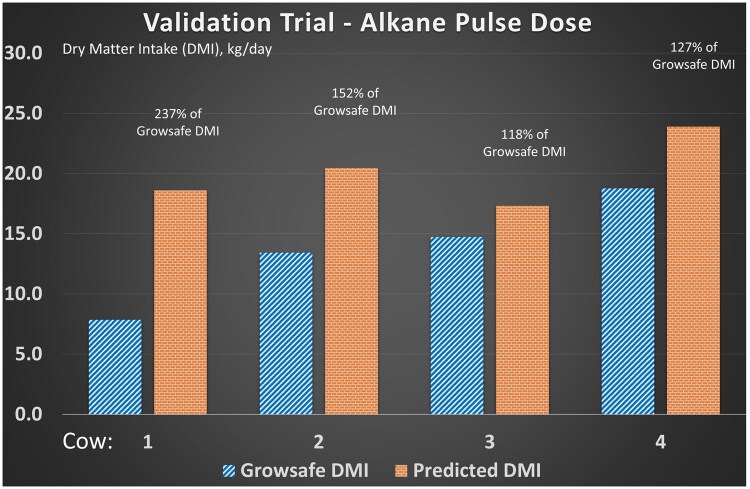
Validation of alkane pulse dose procedure and estimated forage intake against actual feed intake. This feedlot validation trial was conducted using four mature cows in a Growsafe unit. Cows were dosed with alkane and all fecal material was collected for 4 d. Predicted intake was calculated against actual feed intake when the pulse dose was active (0200 on 11 December 2016 to 2300 on 13 December 2016). Cow 1 likely lost some of the alkane when the dose was administered. Based on these data, the predicted intake was 132% of the actual intake for cows 2 to 4.

Figure 2 presents the results of the feedlot validation trial conducted in the GrowSafe unit. The predicted DMI over 3 d was 132% of the actual DMI for cows 2 to 4. Cow # 1 had the greatest divergence between actual and predicted DMI and it is assumed that she lost some of her pulse dose shortly after administration. Therefore, data from this cow was not used to calculate actual DMI.

Fecal recoveries of dosed C_32_ alkanes are usually quite high (89% to 104%, Ohajuruka and Palmquist, 1991; 95%, Giraldez et al., 2004; 95% to 97%, Olivan et al., 2007; 97%, Morais et al., 2011; 93%, Andriarimalala et al., 2020). As such, dosed alkanes can be a reliable external marker to use in grazing studies, provided that the analytical capability of the laboratory performing the alkane analyses meets expectations for repeatability. Some of the advantages of using alkanes as an external marker are 1) the aforementioned recovery rate; 2) the small dose required; and 3) the relatively low background levels of C_32_ contained in many grazed forages (Hu et al., 2014). The chief disadvantages for using dosed alkanes as an external marker are 1) cost of analyses (40 USD/sample for our study); and 2) the complexity of the lab analysis (Dove and Mayes, 2006) which includes 27 steps from start to finish. Consequently, some laboratories have had difficulty in performing the alkane analysis. As previously mentioned, the intra-assay CV averaged 4.11%, which indicates good repeatability of our alkane analyses. Recently, a simplified alkane analysis method has been published which shows some promise for reducing both the cost and time involved in alkane analysis (Liu et al., 2022).

### Nonlinear fitted curves for alkane fecal recovery

**Figure 3.**
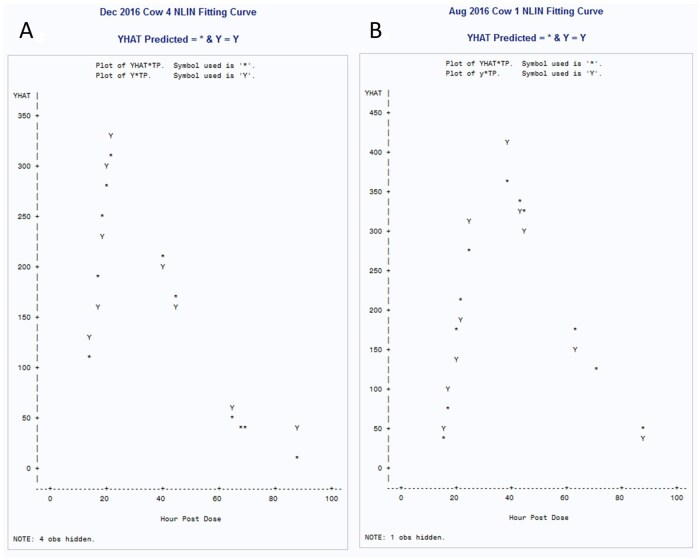
Example fitted nonlinear logarithmic curves for recovered marker concentrations of a pulse dose of C_32_ alkane. The pulse dose was given the afternoon preceding fecal sampling starting at daybreak the following day and continuing over 4 d. The YHAT value is the predicted alkane concentrations from the nonlinear statistical model. The cow (657 kg) sampled in December 2016 (panel A) was housed in a feedlot equipped with a Growsafe device and had an average predicted forage intake (from the alkane marker) of 23.89 kg which was 127% of the actual feed intake (from the Growsafe device) of 18.77 kg. Forage quality for December 2016 for the 100% chopped forage diet was 57.0% total digestible nutrients (TDN) and 17.3% crude protein. Predicted dry matter intake for the cow (469 kg) sampled in August 2016 (panel B) was 15.4 kg. Forage quality for the cow sampled in August 2016 while grazing native rangeland pasture was estimated at 57.1% TDN and 12.3% crude protein from daily fecal samples using near infrared spectroscopy (NIRS). Please note the difference in the peak alkane concentration in the feces for each cow, reflective of rumen fill and forage quality. The predicted time until the first appearance of the alkane marker in the feces for the feedlot cow was 9.94 h and for the rangeland pasture cow was 11.86 h. Four observations were hidden for the December 2016 logarithmic curve and one observation was hidden for the August 2016 curve.

Figure 3 displays fitted nonlinear C_32_ alkane curves for one cow in the validation trial and one cow in the August 2016 trial. Fecal sampling for free ranging cows commenced at daybreak following a mid-afternoon pulse dose of alkanes the day previous. Please note that the alkane pulse dose marker was able to be successfully used in both a higher CP diet (validation trial, 57% TDN, 17.3% CP) and a lower CP rangeland diet (August 2016, 57.1% TDN, 12.4% CP). The peak fecal alkane concentration in the logarithmic curve for the feedlot cow # 4 was around 20 h vs. 40 h for the cow # 1 on rangeland, reflective of greater rumen fill with late season forage. The tau value for cow 4 was 9.94 h vs. 11.86 h for cow 1. Cow 4 was a nonlactating Angus × Hereford cow that weighed 657 kg and her *ad libitum* DMI was 2.9% of body weight. Cow 1 was a 2-yr-old Angus × Hereford lactating cow that weighed 469 kg and her estimated DMI was 3.3% of body weight. The fill (CMS) for the feedlot cow 4 was 10.7 kg vs. 9.2 kg for the rangeland cow 1. The mean square error for the nonlinear alkane fecal prediction curve for August 2016 for Cow 1 was 1,136.6 parts per million (ppm) and for the feedlot alkane prediction curve was 525.5 ppm. The pulse dose alkane marker appeared to function well for both feedlot and rangeland diets, but with some inflation of DMI.

### Background levels of C_32_ alkanes in fecal samples

Background levels of C_32_ alkane in fecal samples prior to pulse dosing of the alkane marker was very low in agreement with published literature (Hu et al., 2014). The average concentration of fecal C_32_ alkane for spring was 6.36 ± 0.68 mg/kg fecal DM (*n *= 24) and for summer it was 2.94 ± 0.53 mg/kg fecal DM (*n *= 23). When expressed as a percentage of the recovered dosed C_32_ in the fecal DM, it was 0.63% in the spring and 0.34% in the summer.

### Climate data

Climate data are shown in Table 1. Maximum and minimum daily temperatures during the June 2016 sample period were slightly cooler than average while temperatures in August 2016 were closer to normal. Sprinkle et al. (2021a) established a relationship between hotter temperatures and daily grazing behavior with these same cattle when cattle moved into mild heat load when the temperature humidity index (THI) met or exceeded 72 (Du Preez et al., 1990; Armstrong, 1994). There were 0 h with THI > 72 in June 2016 but the THI exceeded 72 for 9 h during August 2016.

**Table 1.** Climate data for research trial on Idaho rangeland^1^

Sample period	Average daily maximum temperature, °C	Average daily minimum temperature, °C	Precipitation, mm	Total h THI^a^ ≥ 72
**14 June to 17 June 2016**	18.0	5.5	0	0
**2 August to 5 August 2016**	28.3	9.8	0	9
**Historic averages (June)**	23.6	6.8	41	
**Historic averages (August)**	29.9	9.2	7	

1 Temperature data are from the Freidman Memorial Airport weather station in Hailey, Idaho, 18 km northeast of experimental pastures. Due to missing data, rainfall data are from the Bellevue weather station in Bellevue, Idaho, 14 km northeast of experimental pastures. Long term averages listed are from historic data at the Freidman Memorial Airport (1981 to 2010).

a THI, temperature humidity index. Mild heat load is experienced by livestock when the THI exceeds 72.

### Forage production and quality

**Figure 4.**
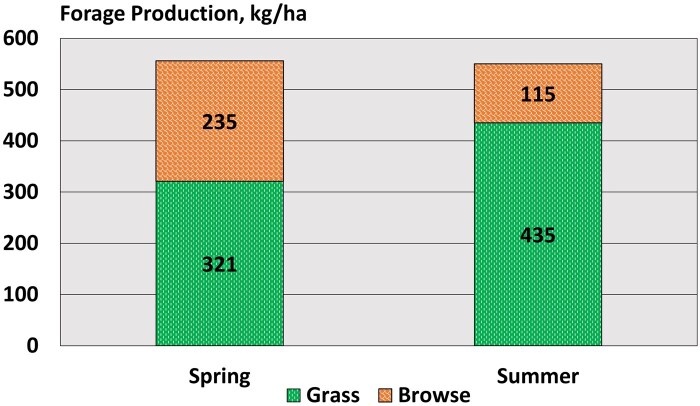
Forage production in experimental pastures at the time of cattle entry. Forage was clipped from 20 randomized plots and separated into grass and browse production. Grass production included both perennial and annual grasses and browse included that year’s production of plant leaders. Most browse production was from sulfur-flower buckwheat. The 90% confidence interval for total forage production in the spring was ± 53 kg and for summer was ± 78 kg.

With forage production exceeding 500 kg/ha in our experimental pastures (Figure 4), the forage allowance for cows in this study was 93.8 kg/animal unit day for the spring sample period and 192.5 kg/animal unit day for the summer sample period. Projecting maximal forage intake to be 3% of body weight (Sprinkle, 2018), we estimated that forage utilization in the spring experimental pasture would be around 15% and for summer, it would be around 7% of available forage. At this slight level of utilization, we did not expect that forage supply would hamper forage intake for the cows in our study.

**Figure 5.**
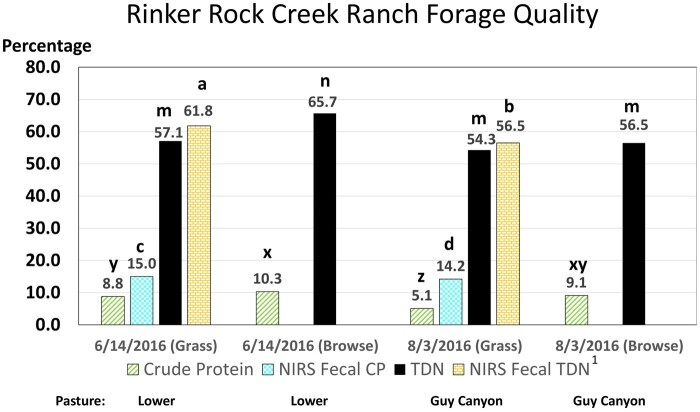
Forage quality of available rangeland forage as estimated from either laboratory analysis of random clipped samples at the time of cattle entry into the pasture or by near infrared spectroscopy (NIRS) analysis of daily fecal samples over 3 d for each cow (less one inefficient cow in spring). There were no differences between Efficient and Inefficient treatment groups for forage quality, but there were differences within the treatment group from spring to summer. The fecal NIRS digestibility declined from spring to summer for both Efficient and Inefficient cows (*P *< 0.0001). For NIRS fecal crude protein, Inefficient cows did not differ (*P *= 0.22) from spring to summer (15.1% ± 0.33% vs. 14.5% ± 0.31%), but the Efficient treatment group had greater NIRS fecal crude protein (15.0% ± 0.31%; *P *< 0.05) in the spring than they did in the summer (13.9% ± 0.31%). Laboratory analyses of forage samples showed similar trends from spring to summer; TDN for spring grass tended (*P *= 0.07) to be greater than in summer. Browse samples generally had higher forage quality (*P *< 0.05) than grass samples. ^1^NIRS digestible organic matter (DOM) was converted to total digestible nutrients (TDN) by multiplying DOM × 1.05 (Huston and Pinchak, 1991). ^a,b^TDN estimated from fecal samples differed between spring and summer (*P *< 0.0001). ^m,n^TDN determined from lab analysis of replicate forage samples differed between spring and summer (*P *< 0.0001). ^c,d^CP estimated from fecal samples differed between spring and summer (*P *< 0.05). ^x,y,z^CP estimated from lab analysis of replicate forage samples differed between spring and summer (*P *< 0.05).

As expected, forage quality declined from spring to summer (Figure 5). Considering the opportunity for animal selectivity for animal-harvested vs. hand clipped forage (Sprinkle et al., 2000), fecal NIRS values for TDN (adjusted from DOM; Huston and Pinchak, 1991) were similar between lab samples and animal harvested values. It should be mentioned that soil contamination of fecal samples could have affected NIRS DOM estimates. The CP for consumed forage (as estimated by fecal NIRS samples) in June had 15.0% CP while randomly harvested hand clipped grass samples had 8.8% CP. Although the selected diet CP is greater, this is not greatly beyond expectations for animal selection for diet quality at this time of year (Grings et al., 2001). The difference between clipped forage and animal harvested forage for CP in August of 2016 was much greater (Figure 5). One factor contributing to this greater difference is that our randomly clipped quadrant samples in August would have contained more dried out grasses, particularly from annual grasses. However, one wonders if the cattle in this study were truly able to compensate for crude protein in the diet as is shown in Figure 5. South and west facing slopes (112.5 to 292.5°) accounted for 51.2% of the experimental pasture, so there was some opportunity for improved diet selection on the more north and east facing slopes. Cows were in the 34-ha experimental pasture in August for only 4 d and with the large forage allowance present they were able to selectively graze for better diet quality. However, DOM values for the NIRS fecal samples did not show evidence for a higher CP diet at this later season of the year. We generally expect greater CP to be highly correlated with greater DOM. It appears that the fecal NIRS DOM values were more in line with the forage available. To predict forage intake, a good estimate of forage digestibility is critical. In this respect, we can express more confidence with DOM than we can for CP estimated by fecal NIRS samples, for this study conducted on sagebrush steppe pastures in Idaho. Perhaps, further evaluation and a region-specific fecal NIRS calibration (Tolleson et al., 2025) for CP is needed for cattle consuming the cool season forage species common to Idaho such as bluebunch wheatgrass, Idaho fescue, Sandberg bluegrass, and great basin wildrye.

When fecal NIRS sampling for forage quality was implemented by Texas A & M University, there was close agreement between predicted and actual DOM and CP for fecal samples from esophageal fistulated steers and their consumed diet (Lyons and Stuth, 1992). Some criticism was levied against the technique for NIRS calibration samples being heavily weighted towards warm season grasses. Consequently, the authors and developers of the NIRS fecal sampling program at TAMU actively sought to expand their library of fecal standards to different parts of the United States and to other countries. They have also engaged in nutritional modeling of cow performance against localized NIRS predictions of diet quality and intake (Tolleson and Schafer, 2014; Brooks et al., 2021; Tolleson et al., 2025).

Returning to a discussion of the NIRS fecal predictions of CP in Figure 5, CP declined slightly from spring to summer (*P *< 0.05), but not for INE cattle (*P *= 0.22). The dietary CP for EFF cattle was greater in spring that it was in the summer (*P *< 0.05). It is our belief that due to the greater nutritional demands for INE cattle, they engage in more search grazing to maintain energy intake as forage resources decline (Sprinkle et al., 2019, 2021a). Gregorini et al. (2015) further reported that lactating EFF Holstein cows walked less and more slowly than did lactating INE Holstein cows as well as taking more bites per feeding station while grazing a common pasture.

The declining DOM from spring to summer shown in Figure 5 for both fecal NIRS and clipped samples provides evidence for the increase in rumen fill and longer residence times that accompany late season rangeland forage.

### Data range for selected dependent variables in the experiment

Since there may be some interest in the range of data we encountered in this experiment, Table 2 presents the same. There is considerable spread to the data, but the means reported are within the realm of possibility for lactating cattle grazing sagebrush steppe rangelands; with some inflation of DMI possibly being caused by the greater recovery of the alkane marker.

**Table 2.** Data range for selected measurements for lactating 2-yr-old cows on Idaho rangeland in 2016

	Spring (14 to 17 June)		Summer (2 to 5 August)	
Item^1^	Minimum	Maximum	Minimum	Maximum
**λ_1_, rate**	0.10	0.89	0.10	0.32
**Passage rate (k_2_), h^-1^**	0.033	0.061	0.036	0.061
**τ, h**	5.5	20.7	7.0	15.8
**CO_1_, ppm**	535	1,677	459	1,432
**CMS, kg**	4.0	12.9	5.1	13.5
**RTG, h**	32.8	54.7	36.9	51.1
**Fecal DM output, kg/d**	3.9	10.9	4.6	9.9
**DMI, kg/d**	10.1	26.7	11.5	23.8
**PBWT, % of BW**	2.11	5.13	2.42	5.49
**EI, kcal ME intake · kg metabolic BW · d^-1^**	173	414	187	417
**ME of consumed OM, Mcal/kg**	2.12	2.31	1.93	2.21
**Grazing, min/d**	531	730	444	722
**Bite rate, bites/min**	26	45	31	48
**HE, g OM intake · kg BW^-1^ · min of grazing**	0.026	0.067	0.029	0.065
**Harvest rate, g DMI/bite**	0.38	1.31	0.42	0.93

1 λ_1_ (lambda), age dependent turnover rate in the first compartment of undigested organic matter; τ, time delay of dosed alkane to first appearance in the feces; CO_1_, is parts per million (ppm) of alkane recovered in feces; CMS, compartmental mass of undigested organic matter in the digestive system (fill); RTG, gastrointestinal tract residence time, 2/λ_1_ + 1/k_2_ + τ; DM, dry matter; DMI, dry matter intake; PBWT, DMI expressed as a percentage of body weight (BW); EI, energy intake; ME, metabolizable energy; OM, organic matter; HE, harvest efficiency. Passage rate values were constrained to a maximum of 0.061 in the nonlinear model discovery.

### Digesta kinetics

#### Lambda

Table 3 contains the results of the alkane pulse dose digesta kinetics analyses. Lambda (λ) reflects the age-dependent rate of escape from the rumination compartment of the two compartment (age-dependent, age-independent) digestive system. As such, it is subject to rate changes as the forage quality declines and requires more time for the breakdown of forage particles. Hence, the lambda values reported in Table 3 reflect the rate change associated with the lower quality forage from spring to summer (*P *< 0.01).

**Table 3.** Digesta kinetics, forage and energy intake, harvesting efficiency and rate, and cow measurements for lactating 2-yr-old cows on Idaho rangeland in 2016

	Period		Spring (14 to 17 June)		Summer (2 to 5 August)	
Item^2^	Spring	Summer	Efficient^1^	Inefficient^1^	Efficient^1^	Inefficient^1^
**λ_1_, rate**	0.33 ± 0.028^a^	0.19 ± 0.028^b^	0.34 ± 0.039^a^	0.32 ± 0.039^a^	0.19 ± 0.039^b^	0.19 ± 0.039^b^
**Passage rate (k_2_), h^-1^**	0.055 ± 0.002	0.055 ± 0.002	0.055 ± 0.002	0.055 ± 0.002	0.055 ± 0.002	0.054 ± 0.002
**τ, h**	12.8 ± 0.56	13.2 ± 0.56	12.7 ± 0.79	12.8 ± 0.79	12.8 ± 0.79	13.6 ± 0.79
**CO_1_, ppm**	1,003 ± 56	856 ± 56	947 ± 80	1,058 ± 80	875 ± 80	838 ± 80
**CMS, kg**	6.6 ± 0.42^a^	8.6 ± 0.42^b^	7.2 ± 0.60^a^ ^,^ ^b^	6.0 ± 0.60^b^	8.4 ± 0.60^a^	8.8 ± 0.60^a^
**RTG, h**	39.4 ± 0.8^b^	43.4 ± 0.8^a^	40.5 ± 1.2^b^ ^,^ ^c^	38.3 ± 1.2^c^	42.9 ± 1.2^a^ ^,^ ^b^	44.0 ± 1.2^a^
**Fecal DM output, kg/d**	6.4 ± 0.31	7.2 ± 0.31	6.7 ± 0.44	6.0 ± 0.44	7.1 ± 0.44	7.4 ± 0.44
**DMI, kg/d**	16.6 ± 0.80	16.6 ± 0.80	17.6 ± 1.10	15.6 ± 1.15	16.3 ± 1.10	17.0 ± 1.10
**PBWT, % of BW**	3.6 ± 0.17	3.6 ± 0.16	3.8 ± 0.23	3.3 ± 0.24	3.6 ± 0.23	3.7 ± 0.23
**Adjusted PBWT, % of BW**	2.7 ± 0.13	2.8 ± 0.13	2.9 ± 0.18	2.5 ± 0.19	2.7 ± 0.18	2.8 ± 0.18
**EI, kcal ME intake · kg metabolic BW · d^-1^**	288 ± 25	271 ± 26	305 ± 35	271 ± 36	263 ± 35	278 ± 39
**ME of consumed OM, Mcal/kg**	2.22 ± 0.014^a^	2.03 ± 0.014^b^	2.22 ± 0.019^a^	2.22 ± 0.020^a^	2.02 ± 0.019^b^	2.03 ± 0.019^b^
**Grazing, min/d**	606 ± 20	643 ± 19	624 ± 27	588 ± 30	647 ± 26	640 ± 27
**Bite rate, bites/min**	36 ± 1.9^b^	39 ± 1.9^a^	35 ± 2.6^b^	36 ± 2.7^a^ ^,^ ^b^	40 ± 2.6^a^	38 ± 2.7^a^ ^,^ ^b^
**HE, g OM intake · kg BW^-1^ · min of grazing^-1^**	0.045 ± 0.004	0.044 ± 0.003	0.043 ± 0.005	0.048 ± 0.005	0.040 ± 0.005	0.048 ± 0.005
**Harvest rate, g DMI/bite**	0.78 ± 0.046	0.67 ± 0.042	0.78 ± 0.063	0.78 ± 0.066	0.62 ± 0.060	0.72 ± 0.060
**Cow weight, kg**	467 ± 7.1	461 ± 7.1	463 ± 10.0	472 ± 10.0	458 ± 10.0	463 ± 10.0
**Cow BCS**	5.0 ± 0.28^a^	5.5 ± 0.28^b^	4.8 ± 0.40^b^ ^,^ ^d^	5.1 ± 0.41^c^ ^,^ ^d^	5.3 ± 0.40^a^ ^,^ ^c^	5.6 ± 0.41^a^ ^,^ ^b^

1 Efficient cows were ranked as low-residual-feed intake and inefficient cows were ranked as high-residual-feed intake as yearling heifers. In the spring of 2016, there were *n *= 12 cows for both efficient and inefficient cows for all measurements except: grazing minutes/d (*n *= 11 both groups); HE and harvest rate (*n *= 11 for efficient; *n *= 10 for inefficient); DMI, PBWT, and EI (*n *= 12 for efficient, *n *= 11 for inefficient). In the summer of 2016, there were *n *= 12 cows for both efficient and inefficient cows for all measurements.

2 λ_1_ (lambda), age dependent turnover rate in the first compartment of undigested organic matter; τ, time delay of dosed alkane to first appearance in the feces; CO_1_, is parts per million (ppm) of alkane recovered in feces; CMS, compartmental mass of undigested organic matter in the digestive system (fill); RTG, gastrointestinal tract residence time, 2/λ_1_ + 1/k_2_ + τ; DM, dry matter; DMI, dry matter intake; PBWT, DMI expressed as a percentage of body weight (BW); Adjusted PBWT, DMI * 0.7639 for overprediction of DMI as noted in validation study; EI, energy intake; ME, metabolizable energy; OM, organic matter; HE, harvest efficiency; BCS, body condition score (1 to 9, 9 = fattest).

a,b,c,d Means within row, by sampling period or sampling period × cow efficiency, with differing superscripts differ (*P *< 0.05).

#### Passage rate

There were no differences in passage rate from spring to summer or among the treatment groups (*P *> 0.77). Passage rate for beef cattle generally varies from 1% to 6% (Owens and Goetsch, 1988), with slower rates being observed for late season dormant forage (e.g. 2.5%/h; Hess et al., 1994) and tropical forages (e.g. 1.29%/h and 2.8%/h; Kennedy et al., 1992). Passage rate increases with greater intake (Okine and Mathison, 1991; Seo et al., 2006) and improved nutritional quality (Owens and Goetsch, 1988). Plants with more soluble cell walls will pass more quickly into the lower tract.

The passage rates reported in Table 3 are comparable to the passage rate observed on an unburned native range pasture in May at El Reno, OK that was dominated by warm season grasses (Scott et al., 1988). Passage rates at that site declined from 5.5%/h in May to 4.4%/h in June and then back up to 4.7%/h in July. The passage rate in our research trial did not decline from spring to summer (*P *= 0.77) as the forage quality declined. Our range sites were heavily dominated by C3 cool season grasses which contain a higher percentage of soluble cell walls than C4 warm season grasses (Wilson et al., 1983; Bohnert et al., 2011). As such, forage digestibility, passage rate, and forage intake do not appear to be depressed as severely for C3 grasses as has been noted for C4 grasses as these forages mature (Bohnert et al., 2011).

#### Tau and marker concentration

The time delay until the marker first appeared in the feces did not differ by sampling period or treatment (Table 3, *P *> 0.53). There was a tendency (*P *= 0.07) for the concentration of the alkane marker (CO_1_) to be greater in the feces during the spring, and this reflects increased ruminal fill (CMS) later in the summer which diluted the dose. This tendency held true for INE cattle (*P *= 0.05), but not EFF cattle (*P *= 0.50). Greater appetites associated with INE cattle likely affected this outcome (Parsons et al., 2020).

#### Rumen fill and total tract residence time

As hemicellulose, cellulose, and lignin increase with advancing plant maturity, plants take longer to be broken down in the rumen by microbial action. Consequently, rumen fill and residence time increase in beef cattle grazing late season forage. Both CMS (*P *= 0.0022) and RTG (*P *= 0.0005) increased from spring to summer for the cows in this study (Table 3). Although there were no differences among the treatment groups for either CMS (*P *= 0.48) or RTG (*P *= 0.65), there was a difference in how EFF and INE cattle responded to the advancing season. There was no difference in CMS from spring to summer for EFF cattle (*P *= 0.15), but INE cattle increased in CMS from spring to summer (*P *= 0.003). The RTG tended (*P *= 0.097) to increase for EFF cattle but was significantly greater for INE cattle (*P *= 0.0005).

It has been proposed that INE cattle may have greater CMS due to their increased appetite (Sprinkle et al., 2021a). Research has clearly demonstrated longer meal duration and more frequent meals/day for feedlot steers fed a concentrate diet (Parsons et al., 2020). The cattle used in our study were also part of an expanded grazing behavior study at RRCR in 2016 to 2017 (Sprinkle et al., 2021a). Based upon earlier work done in Texas (Sprinkle et al., 2000) and Ireland (Fitzsimons et al. 2014), the authors of the Sprinkle et al. (2021a) study implicated larger digestive tract size as a probable link to increased metabolic heat load for INE cattle in August 2016. When experiencing mild heat load at THI ≥ 72, INE cattle started grazing later during the afternoon and favored lower elevation areas of the pasture than did EFF cattle. Our current research confirms the hypothesis that the increased heat stress of the same INE cattle during the summer of 2016 may have indeed been related to increased CMS from spring to summer.

#### Fecal output

Fecal output tended to increase (Table 3) from spring to summer (*P *= 0.08) and for INE cattle from spring to summer (*P *= 0.06), but not EFF cattle (*P *= 0.59). These data are a mirror that reflects the same trends noted for CMS. The size of the gastrointestinal tract is directly related to appetite, forage quality, fecal output, and forage intake.

### Forage and energy intake

There were no differences in DMI (*P *> 0.50) or EI (*P *> 0.42) by either sampling period or treatment group. With respect to DMI for cattle differing in RFI, our findings agree with most of the limited studies done with these divergent groups of cattle on grazing lands (Herd et al., 1998; Lawrence et al., 2012, 2013). We have only found one study which showed a difference for DMI between efficient and inefficient grazing cattle (Manafiazar et al., 2015). We subscribe to the conclusions reached by Lawrence et al. (2012, 2013) that different feed intake mechanisms such as ruminal fill may exist for cows grazing forage-based diets when compared to cattle in a feedlot setting.

A weakness of our study is that our DMI is only a snapshot in time for one value over a 4-d period. Livestock exhibit a great deal of variation in intake, even within a feedlot (Galyean and Gunter, 2016). The CV for the 4 cows in our validation trial for daily DMI over 5 d was 20.1%. In a series of cattle grazing behavior studies we have conducted, we found that daily grazing time has varied from 6.5 to 12.4 h/d (Sprinkle et al., 2021a, 2021c; and unpublished data). Since forage intake/d can be calculated as bites/grazing min × grazing min/d × g DMI/bite (Arnold and Dudzinski, 1978), considerable variation in consumed forage can be expected as daily grazing time changes. Although it would have been advantageous to have obtained forage intake for each day over a 10-d period, no constant release device was available at the time of our rangeland study. Furthermore, we would have been unable to obtain digesta kinetics data and we would have failed to establish the linkage of INE cattle heat stress to increased CMS in late summer.

Since grazing cattle will adjust intake over time to meet energetic needs (Galyean and Gunter, 2016), it makes sense to define cow maintenance requirements in terms of calories consumed. Despite decreasing ME (*P *< 0.0001) in the consumed forage from spring to summer (Table 3), the cattle in this study were effective in maintaining EI at over 200 kcal/kg^0.75^ which Havstad and Doornbos (1987) defined as being adequate for maintenance for lactating grazing cattle. Ferrell and Jenkins (1984) reported that the maintenance requirement for a nonlactating, non-gestating Angus × Hereford cow in the feedlot was 140 kcal/kg^0.75^. If we presume the activity adjustment for grazing to be a multiplier of 1.3 (Osuji, 1974), then the maintenance requirements for a non-lactating 461 kg Angus × Hereford cow in our study would be 18.2 Mcal ME/d. Milk production estimated by weigh-suckle-weigh procedures in August 2016 for these experimental cows averaged 5.1 kg (Sprinkle et al., 2021a). Assigning a value of 1.06 Mcal of ME per kg of milk production (3.5% butterfat; NASEM, 2021) would add another 5.4 Mcal of ME to the maintenance requirements, for a total of 23.6 Mcal of ME required per day. The cows in our study consumed 271 kcal/kg^0.75^ (Table 3) in summer, or a total of 26.9 Mcal of ME, which was 14% above the predicted maintenance requirement. If we use the increase in maintenance for milk production of 9.6 kcal/cow kg^0.75^ for each kg of additional milk production (at peak lactation) reported by Ferrell and Jenkins (1987) and the activity adjustment of Osuji (1974) then maintenance requirements for our cows would be estimated at 23.1 Mcal ME in August. This would place actual EI at 16% above the predicted intake. Although we identified a good range of data for EI (Table 2), our overall means agree with EI predictions and values from reported literature.

When our reported forage intake means were expressed as a percentage of body weight (Table 3), no differences among treatment groups or by sampling period were detected (*P *> 0.32) and our means exceeded the “rule of thumb” values for forage intake suggested (2.2% to 2.7%) by Lalman (2004). If percentage of body weight intake had been expressed on an OM basis, then percentages would have ranged from 2.60% to 3.01% (with fecal ash limited to 15%) for the cows in our study.

To compensate for the possible overprediction of DMI by the cows in our study using the alkane marker, we present adjusted percentage of body weight intakes in Table 3. These means are adjusted downwards by a multiplication factor of 0.7639 in line with our validation study. Doing so reduced the percentage of body weight intake means to a range of 2.5% to 2.9%.

### Grazing behavior, harvesting rate, and harvesting efficiency

#### Grazing time and bite rate

There was a tendency (*P *= 0.06) for grazing time to increase from spring to summer for all cows in the study and particularly for INE cattle (*P *= 0.07). The EFF cattle did not differ (*P *= 0.41) for grazing time from spring to summer, rather they increased (*P *< 0.05) their bite rate instead. The bite rate also increased for all cows combined from spring to summer (*P *< 0.05), but not for INE cattle (*P *= 0.52). Based upon the preponderance of data we and others have collected (Gregorini et al., 2015; Sprinkle et al., 2019, 2021a); we believe that there are differences in how EFF vs. INE cattle graze. We have concluded that INE cattle engage in more search grazing to accommodate dietary needs while EFF cattle appear to be more “purposeful” in how they graze.

#### Harvesting efficiency and harvest rate

The harvesting efficiency reported in Table 3 did not differ by either sampling period or treatment group (*P *> 0.32). Similarly, harvest rate did not differ as well (*P *> 0.10). With the ample forage allowance available to the cattle in this study, the harvest rate exceeded 0.6 g/bite. According to Bailey et al. (1996), with a bite size of 0.62 g/bite, a 400 kg herbivore should be able to consume forage at 75% of the maximum rate of intake. As the bite size increases to 1.0 g/bite, intake starts to plateau at 82% of maximal intake.

### Cow measurements

There was a tendency (*P *= 0.08) for cow weights to be greater in spring than in summer, but this may reflect the 4.5 h additional shrink time in August before cattle were weighed. Accounting for 1%/h shrink for the first 3 to 4 h and then 0.25%/h for the next 8 to 10 h (Parish and Rinehart, 2021) would remove any tendency for differences in weights by sample period or RFI treatment (*P *= 0.086 for INE cows; *P *= 0.43 for EFF cows). Cow BCS was greater in the summer than it was in the spring (*P *= 0.0005) and there was no difference among treatment groups (*P *= 0.58). Both INE and EFF cattle gained 0.5 BCS from spring summer (*P *< 0.01), indicating adequate forage quality and quantity on these rangeland pastures.

## Conclusions

Only one of the grazing studies for forage intake we have found comparing efficient (low-RFI) vs. inefficient (high-RFI) cattle reported a difference between the treatment groups (Manafiazar et al., 2015). That study estimated intake over 5 d. The other studies we have evaluated for estimating intake for divergently ranked RFI cattle occurred over fewer days (Herd et al., 1998 [3 d]; Herd et al., 2002 (4 d); Lawrence et al., 2012 (1 d); Lawrence et al., 2013 (1 d); Oliveira et al., 2018 (3 d)]. Considerable day to day variation in forage intake can be expected with free ranging livestock grazing extensive rangeland pastures. It would be advantageous to obtain forage intake estimates over longer time periods but there are currently no good options for doing so without unduly disturbing the experimental animals. With small, improved pasture paddocks less than 6 ha in size, daily dosing of an external marker using “tame” experimental animals may be achieved with minimal disturbance. Performing the same daily dose procedures for free ranging cattle grazing larger rangeland pastures in the Western US, Canada, Australia, and other areas of the world is problematic, if not impossible. Yet, these athletic and acclimated cattle are the very ones for which improved estimates of forage intake and energetic demands are lacking (Caton and Olson, 2016). Currently published prediction equations for DMI and the energetic demands of grazing and livestock production on more extensive cattle operations are inadequate (Lardy et al., 2004; Coleman et al., 2014; Caton and Olson, 2016; NASEM, 2016). One method proposed by Coleman et al. (2014) to assist in determining the maintenance requirements for grazing animals would be to feed a composite rangeland forage diet in the feedlot with GrowSafe or similar technology then place the same animals on the rangeland pasture and determine intake with an external marker.

Energy intakes as predicted by the pulse dose of alkanes in this study were aligned with traditional predictions used by range livestock nutritionists, though slightly inflated by 14%. As mentioned previously, our forage intake estimates were short term, a weakness of many grazing studies. Although we were unable to provide conclusive evidence of adaptive grazing responses pursued by EFF vs. INE cattle, we did find hints of the same with the digesta kinetics we observed. Lactating cattle have been shown to have “plasticity” with the size of the gastrointestinal tract, increasing in response to lactational demands, forage supply, and forage quality (Tulloh, 1966; Forbes, 1986). Cattle with greater appetite (such as INE cattle) should have the propensity to increase their gastrointestinal tract size more than EFF cattle as forage quality declines and digestive fill increases. In this study, we documented that the CMS for INE cattle markedly increased from spring to summer, while EFF cattle did not differ. Fecal output, tau, and RTG were connected mathematically to CMS and had a similar response from spring to summer. The physiological penalty of greater CMS for INE cattle in this study has been implicated as a causal factor for increased heat stress (Sprinkle et al., 2021a); resulting in reduced use of steeper terrain and increasing loafing during the heat of the day.

The way nutrients were acquired from spring to summer also differed between EFF and INE cattle. The INE cattle in this study tended to increase grazing time from spring to summer and their intake of CP did not differ. Paradoxically, EFF cattle did not increase their grazing time from spring to summer, rather they ate faster. They also appeared to be more accommodating to the decline in CP from spring to summer. The behaviors exhibited by the INE cattle in this study, coupled with other studies (Sprinkle et al., 2021a, 2021c) demonstrate that INE cattle engage in more search grazing to try to satisfy their increased nutritional demand. Sprinkle et al. (2020) reported that high-RFI cattle lost more weight than low-RFI cattle while grazing late season dormant forage. These and other results in the literature (Herd et al., 1998; Marin et al., 2024) imply that the maintenance requirements for grazing INE cattle appear to be greater than for EFF cattle, though it may be difficult to detect with short term forage intake studies. Improved nutrient partitioning to retained energy for EFF cattle is similarly suggested from the above studies. Marin et al. (2024) used a heart rate-O_2_ pulse method (Brosh, 2007) to determine residual heat production for grazing Hereford heifers sired by bulls differing in RFI (low, medium, high). Heifers sired by low-RFI bulls appear to be more efficient in depositing protein and in maintaining it after it is deposited, suggesting decreased protein turnover (Marin et al., 2024). Some of the major contributions to improved feed efficiency as proposed by Richardson and Herd (2004) include protein turnover, tissue metabolism, and stress (37%); digestibility (10%); activity (10%), and heat increment of fermentation (9%).

The digestibility estimate by fecal NIRS was similar to the chemical lab analysis of forage but fecal NIRS CP may not be truly representative of available herbage for cool season dominated forages in Idaho.

In summary, the alkane pulse dose procedure provides reasonable or slightly inflated estimates of DMI but fails to capture the day-to-day variability in forage intake accompanying range livestock production. Continued efforts should be made to evaluate the efficiency of cattle grazing rangeland along with methods to provide long term daily estimates of energetic demands for grazing livestock.

## Abbreviations:

λ1: lambda is escape rate from 1st mixing compartment for forage particles
τ: time delay of alkane marker until first appearance in feces
BCS: body condition score
BR: bite rate
BW: body weight
C2: concentration that alkane marker achieves as result of instantaneous mixing and dilution by mass of digesta in the slower turnover compartment
CF2: flux of undigested organic matter from CM2
CM1: compartmental mass of undigested OM in the age-dependent first compartment of the digestive system
CM2: compartmental mass of undigested OM in the age-independent second compartment of the digestive system
CMS: CM1 + CM2 of 2-compartment mass of undigested OM (g)
CO1: C2 expressed as parts per million (ppm)
CP: crude protein
DM: dry matter
DMI: dry matter intake
DOM: Digestible organic matter
EFF: efficient
EI: energy intake
GPS: global positioning system
INE: inefficient
k2: passage rate from the rumen
kcal: kilocalories
MCRT1: residence time of alkane marker in the slower turnover mixing compartment (CM2) as calculated by 1/k2
MCRT2: residence time of alkane marker in the faster turnover mixing compartment (CM1) as calculated by 2/λ1
MCRTS: MCRT1 + MCRT2
ME: metabolizable energy
NIRS: near infrared spectroscopy
NMCREEC: Nancy M. Cummings Research, Extension & Education Center
OM: organic matter
PBWT: DMI expressed as a percentage of BW
ppm: parts per million
RFI: residual feed intake
RRCR: Rinker Rock Creek Ranch
RTG: residence time of alkane marker in gastrointestinal tract as calculated by MCRTS + τ
TDN: total digestible nutrients
THI: temperature humidity index
USD: United States Dollar
